# Potentiometric Hydrogen Sensor with 3D-Printed BaCe_0.6_Zr_0.3_Y_0.1_O_3-α_ Electrolyte for High-Temperature Applications

**DOI:** 10.3390/s22249707

**Published:** 2022-12-11

**Authors:** Antonio Hinojo, Enric Lujan, Marc Nel-lo, Jordi Abella, Sergi Colominas

**Affiliations:** Electrochemical Methods Laboratory—Analytical and Applied Chemistry Department, IQS School of Engineering, Universitat Ramon Llull, Via Augusta 390, 08017 Barcelona, Spain

**Keywords:** ceramic 3D printing, potentiometric sensor, BaCe_0.6_Zr_0.3_Y_0.1_O_3-α_, BCZY, perovskite, proton-conducting materials

## Abstract

Hydrogen is expected to play an important role in the near future in the transition to a net-zero economy. Therefore, the development of new in situ and real-time analytical tools able to quantify hydrogen at high temperatures is required for future applications. Potentiometric sensors based on perovskite-structured solid-state electrolytes can be a good option for H_2_ monitoring. Nevertheless, the geometry of the sensor should be designed according to the specific necessities of each technological field. Conventional shaping processes need several iterations of green shaping and machining to achieve a good result. In contrast, 3D printing methods stand out from conventional ones since they simplify the creation of prototypes, reducing the cost and the number of iterations needed for the obtainment of the final design. In the present work, BaCe_0.6_Zr_0.3_Y_0.1_O_3-α_ (BCZY) was used as a proton-conducting electrolyte for potentiometric sensors construction. Two different shapes were tested for the sensors’ electrolyte: pellets (BCZY-Pellet) and crucibles (BCZY-Crucible). Ceramics were shaped using extrusion-based 3D printing. Finally, parameters, such as sensitivity, response time, recovery time and the limit of detection and accuracy, were evaluated for both types of sensors (BCZY-Pellet and BCZY-Crucible) at 500 °C.

## 1. Introduction

Hydrogen is expected to play an important role in the near future for the transition to a net-zero economy, even in sectors where the transition is challenging, such as heavy industry, long-distance transport, chemicals and energy production [1,2]. Production of sustainable hydrogen has been historically difficult. The system used so far consists of steam reforming, in which methane reacts with steam under high pressure, producing significant carbon emissions [3,4]. 

However, recent technological advances and new public and private investments make hydrogen the leading option for storing energy from renewable sources (green hydrogen) and using it as an energy carrier [4]. Tools, such as solid oxide fuel cells (SOFC), are currently under development for the reprocessing of hydrogen into energy [5,6]. Quantification of hydrogen in the fuel stream of these devices can improve their performance, efficiency and lifetime [7]. However, hydrogen will not only be an energy carrier, but it will also be an energy source in future nuclear fusion reactors. These devices will use deuterium and tritium as plasma fuel [8,9]. Since tritium is an unstable hydrogen isotope, it will be generated in situ through a lithium nuclear reaction that will take place at temperatures around approximately 500 °C [9,10]. The monitoring of the generated tritium is required to ensure the correct operation of the reactor. Therefore, the sensors required for hydrogen monitoring in these environments should operate in real-time at high temperatures, with minimally invasive designs that do not disturb the expected performance of each type of application. Different electrochemical sensors can be used to perform hydrogen measurements, such as resistive, amperometric and potentiometric sensors. Note that the last two are based on solid-state electrolytes [11]. Resistive hydrogen sensors typically operate between room temperature and 400 °C [12]. However, this type of sensor needs initial exposure to oxygen, typically from the air, due to its working principle. Briefly, for example, the change in the metal–oxide resistance results from a gain of surface electrons following the reaction of hydrogen with adsorbed oxygen [13]. This initial step limits their application for some fields, such as future fusion reactors. In contrast, electrochemical sensors based on solid-state electrolytes can operate at high temperatures, typically up to 600 °C. Moreover, some of them are reported to be able to work up to 1300 °C [14]. Amperometric sensors are based on the measurement of a current by applying a constant potential between electrodes. Then, the flowing current depends on the hydrogen concentration. The response of these sensors is highly dependent on the active area of the electrodes. In contrast, potentiometric sensors are not dependent on the area, so their response is not expected to change significantly by miniaturization.

Among all solid-state electrolytes, perovskite ceramics present high protonic conductivity [15,16] and good chemical and physical stabilities [17]. In previous works [18,19], BaCe_0.6_Zr_0.3_Y_0.1_O_3-α_ perovskite material was used as an electrolyte for potentiometric and amperometric hydrogen sensors construction. In those works, the perovskite material was pellet-shaped using uniaxial pressure. This method was simple and affordable, but the obtained geometry was very limited for some applications, such as SOFC, which need specific designs due to their space limitation. For that, new geometries should be studied. 

Prototyping of ceramics with complex geometries can be performed with multiple technologies, such as slip casting [20] or cold isostatic pressure (CIP) [21]. However, these traditional processes need several iterations of green shaping, including the design and fabrication of molds and machining of the green body to achieve a good final result. These conventional prototyping processes are time-consuming and cost-intensive due to several iterations, especially for the obtainment of prototypes before arriving at the final design [22]. Alternatively, 3D printing is a simple and versatile technology for shaping almost any type of geometry [23,24]. This technology provides high flexibility and quick prototyping, considerably reducing the cost and number of iterations needed for the obtainment of the final design [25,26]. 

In the present work, BaCe_0.6_Zr_0.3_Y_0.1_O_3-α_ solid-state electrolyte was used for potentiometric sensors construction. This material was selected due to its good chemical stability and high ionic conductivity [27,28,29]. It is worth mentioning that these types of materials are typically pellet-shaped using uniaxial pressure [30,31,32]. As an alternative, the present work describes the shaping of this material using 3D printing. This technology is useful for quick prototyping of small batches and can help to design specific solutions for different industrial needs, such as in SOFC or fusion reactors. However, very little bibliographic data for the 3D printing of these materials is available. For that, in the present work, a BaCe_0.6_Zr_0.3_Y_0.1_O_3-α_ slurry was formulated, and the printing conditions for this slurry were determined. Two different geometries were tested: pellets and crucibles. On the one hand, pellets were selected due to their simplicity and because they can be obtained by methods such as uniaxial pressure. On the other hand, crucibles were chosen because that shape brings advantages, such as easier binding and greater robustness [29]. 

In this work, the ceramic slurry development and the 3D printing conditions are discussed. After that, the construction of the sensors and the experimental setup where sensors are tested are described. Then, in the results and discussion section, the characterization of the samples is detailed. Finally, sintered ceramics were used as an electrolyte in potentiometric sensors, and their response was evaluated and compared at 500 °C. The evaluated parameters for both sensors were sensitivity, response and recovery time and the limit of detection and accuracy.

## 2. Materials and Methods

### 2.1. Shaping with 3D Printing

The BaCe_0.6_Zr_0.3_Y_0.1_O_3-α_ (identification code: BCZY) proton-conducting ceramic was synthesized by a solid-state reaction, as described in previous works [18], with a d_50_ of 1.9 µm. After the synthesis, the ceramic powder was shaped using a commercial 3D printer. Two different shapes were evaluated in the present work: pellets (Ø13 and 1.2 mm thickness; identification code: BCZY-Pellet) and crucibles (Ø13 and 35 mm height and 1.2 mm thickness; identification code: BCZY-Crucible).

**Figure 1:**
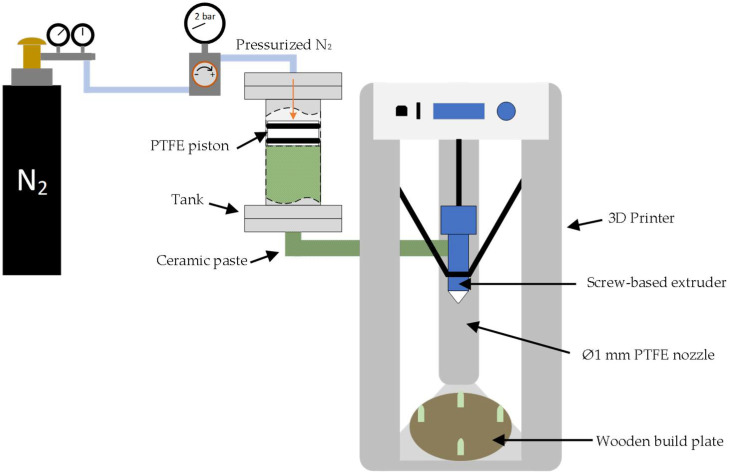
Schematic representation of the experimental setup used for 3D printing.

The 3D printer (Delta Wasp 2040 Clay, Massa Lombarda (RA)—Italy) used for shaping the ceramic powder consists of a screw-based extruder. For that, a high-solid-loading BCZY slurry (83 wt.%) was prepared by mixing the BCZY powder with polyethylene glycol 400 (PEG-400, synthesis grade, Scharlau, Barcelona—Spain) and deionized water in a rotatory ball mill for 1 h, in a ratio of 12:1.5:1 (expressed as weight), respectively. Figure 1 shows a schematic representation of the experimental setup for 3D printing.

As can be observed in Figure 1, the ceramic slurry was placed inside a custom-made stainless-steel tank of 500 cm^3^ volume. The tank was pressurized at 2 bar with nitrogen to move a PTFE piston and push the BCZY slurry to the screw-based extruder. Finally, the slurry was extruded into a wooden build plate using a custom-made 1 mm PTFE nozzle. The printing process was performed at room temperature. In Table 1, the parameters used for the additive manufacturing (AM) process are presented. 

As can be observed in Table 1, the layer height, which is the distance between the different layers printed during the process, was 0.5 mm to ensure good contact between the different printed layers. The print speed and tank pressure were established at 100 mm/s and 2 bar to ensure that the slurry was satisfactorily extruded through the nozzle. The thickness of BCZY-Pellets and BCZY-Crucibles was established at 1.2 mm to prevent cracks during the drying, debinding and sintering processes. For BCZY-Pellet, a zig-zag infill pattern was used. In contrast, BCZY-Crucibles were printed with a spiralized contour. Both printing patterns were selected to ensure a continuous strand during the printing process. The green bodies were dried at room temperature overnight. 

### 2.2. Sintering and Characterization of the Solid-State Electrolytes

Dried green bodies were heated at 1700 °C for 1 h at 5 °C/min for debinding and sintering. The bulk density of the sintered ceramics was calculated using the Archimedes principle. 

Crystallographic phases after sintering were determined using X-Ray diffraction (XRD). The instrument used was a Malvern Panalytical Empyrean (Malvern—UK) using Cu Kα radiation. Crucible-shaped samples were analyzed by grinding the sample after sintering in order to ensure a flat surface. The surfaces of the ceramics were also studied by Scanning Electron Microscopy (SEM). SEM micrographs were obtained using a JEOL JSM-5310 (Tokyo—Japan). 

### 2.3. Potentiometric Sensor Construction

**Figure 2:**
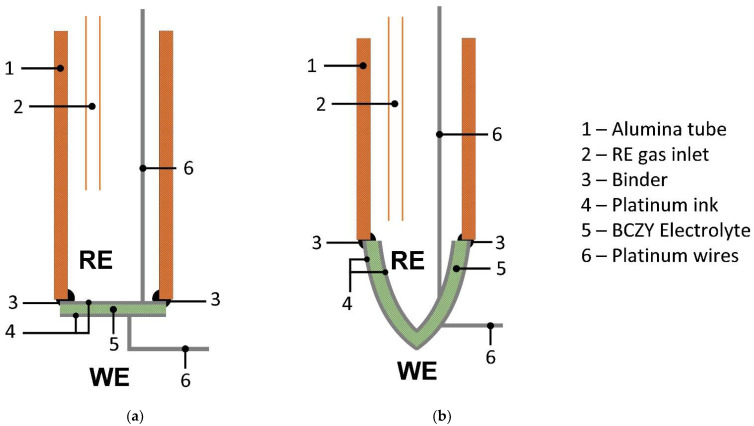
Schematic representation of the potentiometric sensors constructed with BCZY electrolyte shaped as (**a**) BCZY-Pellet and (**b**) BCZY-Crucible. WE: working electrode; RE: reference electrode.

After sintering, each face of the 3D-printed ceramics was homogeneously platinized using platinum ink for ceramic substrates (Alfa Aesar, Massachusetts—USA). Then, Pt-coated ceramics were calcined at 1000 °C for 30 min. Sensors were constructed by binding each BCZY ceramic to alumina tubes (Al-23 Tube, OD 15 mm, ID 11 mm, Alfa Aesar, Massachusetts—USA) using a borosilicate glass cement dispersed in water. Finally, the binder was cured at 900 °C for 1 h. Figure 2 shows a schematic representation of the sensors. 

The external face of the sensors (Figure 2) acted as the working electrode (WE) and the inner chamber as the reference electrode (RE). Thus, the assembly worked as a concentration cell, which is described by the Nernst equation,

(1) $$\Delta E=-\frac{\mathrm{RT}}{\mathrm{nF}}\ln\left(\frac{{\left(P_{H_{2}}\right)}_{\mathrm{WE}}}{{\left(P_{H_{2}}\right)}_{\mathrm{RE}}}\right)$$

where R is the universal gas constant (8.314 J·K^−1^·mol^−1^), n is the number of electrons in the electrochemical reaction, F is Faraday’s constant (96,485 C/mol), and $P_{H_{2}}$ indicates the hydrogen partial pressure at the WE and RE. 

### 2.4. Electrochemical Measurements

**Figure 3:**
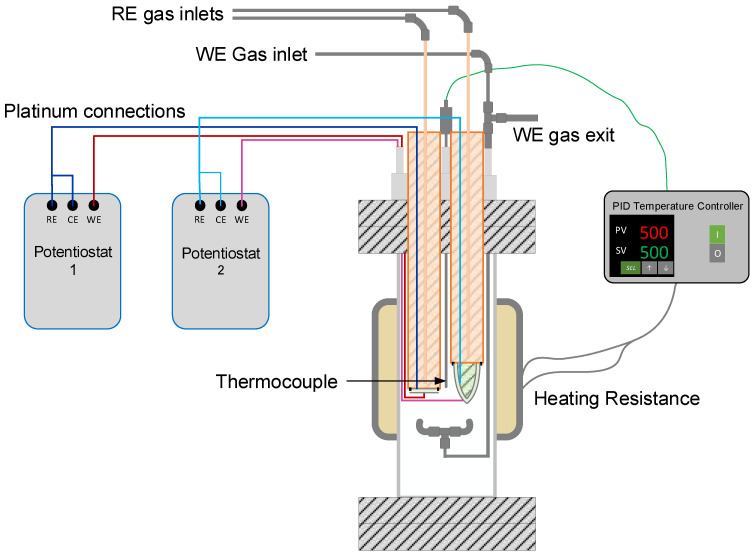
Schematic representation of the experimental setup for electrochemical measurements. WE: working electrode; RE: reference electrode; CE: counter-electrode; PV: process value; SV: set value; and PID: proportional-integral-derivative.

Potentiometric measurements were performed in a stainless-steel reactor. A schematic representation of the experimental setup is shown in Figure 3.

Sensors were placed in a stainless-steel reactor using feedthroughs to prevent gas leakages (see Figure 3). The temperature was controlled using a clamp-type electrical resistance (1500 W) and a thermocouple (type K) connected to a PID temperature controller (Fuji PXR4). The thermocouple was located as close as possible to the outer side of both electrolytes, and it was assumed as the temperature of the WE for both sensors. The reactor was isolated using glass wool (Kaowool^®^ Blanket, Morgan thermal ceramics, Windsor, UK) to prevent temperature fluctuations.

The hydrogen concentration in the reactor (working electrodes) ranged from 0.02 to 0.5 mbar H_2_ in Ar by mixing different flow rates of high-purity argon (99.9992%) and a hydrogen calibration mixture (0.1% H_2_ in Ar), both supplied by Carburos Metálicos (Cornellà de Llobregat—Spain). A calibration mixture of 0.1% H_2_ in Ar (1 mbar) was used as reference electrodes. Flow rates were controlled using gas mass flow controllers (Bronkhorst EL-FLOW, Ruurlo—Netherlands). Finally, the electrodes were connected to a high-impedance voltmeter (PalmSens EmStat3+ Blue) using platinum wires to measure the potential difference (ΔE) at 500 °C.

## 3. Results and Discussion

### 3.1. Characterization of the Electrolytes

**Figure 4:**
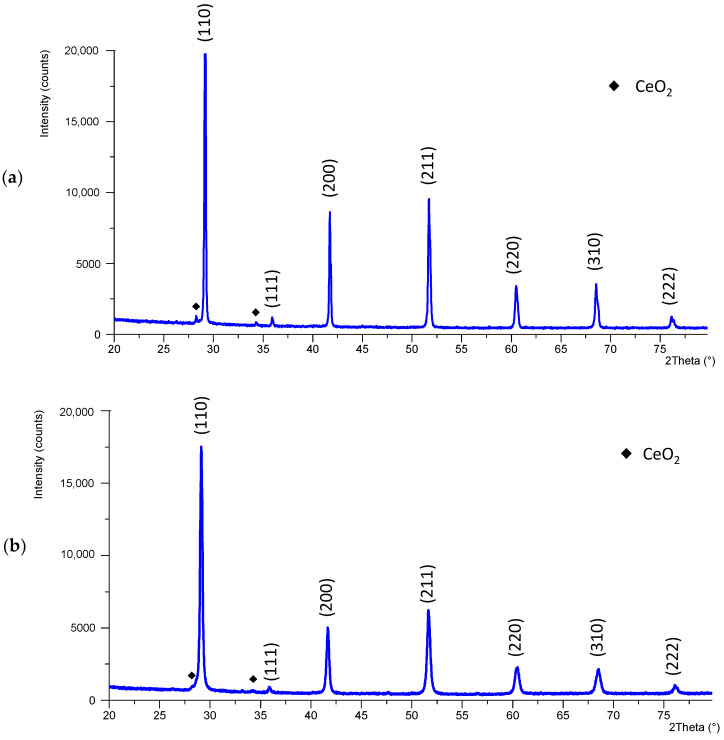
X-ray diffractogram of BCZY pieces after sintering (**a**) BCZY-Pellet and (**b**) BCZY-Crucible.

In Figure S1, an image of the sintered samples can be observed. The crystallographic structure of BCZY ceramics after sintering was analyzed using XRD. Figure 4 shows the XRD patterns obtained for both electrolytes. 

Figure 4 shows that a single cubic perovskite phase was obtained for both electrolytes (ICDD: 04-017-6645, space group Pm-3m). The obtained pattern matched with bibliographic data [28]. However, in both patterns, a few small new peaks were detected after sintering (indicated with **♦** in Figure 4), which corresponded to CeO_2_ (ICDD: 04-013-4458). The patterns were Rietveld refined. For BCZY-Pellet, 1.1% of CeO_2_ was obtained. For BCZY-Crucible, 0.5% was obtained. Thus, it was not expected to influence the sensors’ performance significantly. The lattice parameter was calculated using XRD data for both patterns, which was 4.327 Å for BCZY-Pellet and 4.333 Å for BCZY-Crucible. The theoretical lattice parameter was 4.330 Å (ICDD: 04-017-6645). Thus, the discrepancies with the theoretical ones were 0.07%. According to this, there was no observed barium volatilization during sintering [17,33]. 

**Figure 5:**
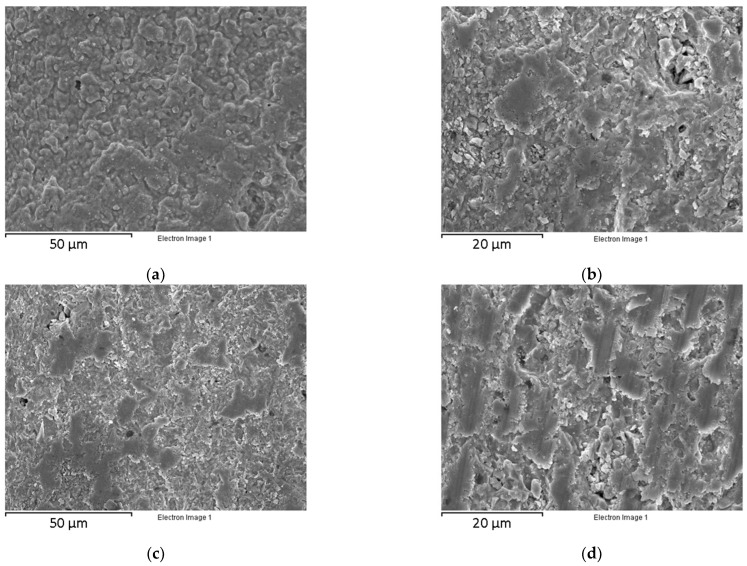
Micrographs of the BCZY 3D-printed pieces after sintering: (**a**) BCZY-Pellet surface, (**b**) BCZY-Pellet cross-section, (**c**) BCZY-Crucible surface and (**d**) BCZY-Crucible cross-section.

The surface and cross-section of the BCZY-Pellets and BCZY-Crucibles were analyzed using SEM (Figure 5). 

A dense surface was obtained without cracks and imperfections for BCZY-Pellets (see Figure 5a). In the cross-section micrograph shown in Figure 5b, good bonding between the printing layers was observed. No crossing channels that could affect the gas tightness were observed. The bulk density of BCZY-Pellets, measured using the Archimedes principle, was (6.10 ± 0.04) g/cm^3^. The theoretical density of BCZY is 6.23 g/cm^3^ [28]. Thus, the densification achieved after the sintering process was (97.9 ± 0.6) %. BCZY-Crucibles surface (Figure 5c) also showed a dense surface without imperfections. The cross-section micrographs of these ceramics (Figure 5d) showed good bonding between the 3D-printed layers. No cracks or crossing channels were observed. The bulk density of BCZY-Crucibles was (5.96 ± 0.05) g/cm^3^ (densification of (95.7 ± 0.8) %). The densification obtained with both electrolytes is high enough (>93%) for completely gas-tight devices [34]. In general, both ceramics were dense and compact and without inconsistencies in the surface and cross-section.

### 3.2. Sensors’ Performance

**Figure 6:**
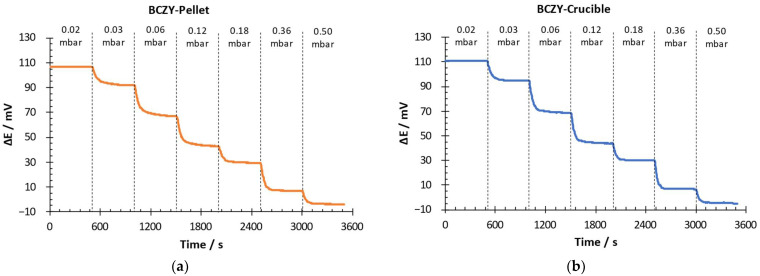
Potential difference-over-time measurement for (**a**) BCZY-Pellet and (**b**) BCZY-Crucible.

The electrochemical performance of the sensors (BCZY-Pellet and BCZY-Crucible) was evaluated at 500 °C in potentiometric configuration. Hydrogen partial pressure was changed from 0.02 to 0.5 mbar of H_2_ in Ar. In the RE, 1 mbar of H_2_ in Ar was used. Figure 6 shows the potential difference (∆E) over time at each hydrogen partial pressure. 

Both sensors quickly detected changes in the hydrogen partial pressure in the WE. The potential difference measured with both sensors decreased when the H_2_ partial pressure increased. This behaviour was in good agreement with the Nernst equation (Equation (1)). It can also be observed in Figure 6 that the signal oscillation after stabilization was lower than 1 mV for both sensors. 

**Figure 7:**
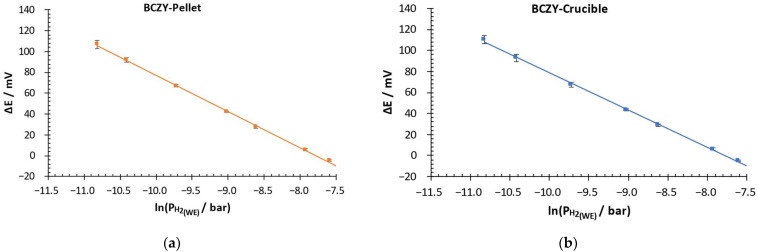
Potential difference over the logarithm of the H_2_ partial pressure in the WE of (**a**) BCZY-Pellet and (**b**) BCZY-Crucible sensors.

With the aim of analyzing the performance of the sensors in more detail, the potential difference was plotted as a function of the logarithm of the partial pressure in the WE for both sensors. Measurements were performed with three BCZY-Pellet and three BCZY-Crucible sensors. Figure 7 shows the obtained calibration curves. 

Both sensors showed a linear response between 0.02 and 0.5 mbar and good repeatability. The standard deviations obtained between measurements were the following: for BCZY-Pellet sensors, 0.6–4 mV, and for BCZY-Crucible sensors, 0.5–4 mV.

Table 2 shows the theoretical calibration curve calculated using the Nernst equation and the calibration curves and limit of detection (LOD) obtained with each type of sensor. 

As can be observed in Table 2, both sensors presented a linear relationship between the potential difference and the logarithm of the partial pressure in the evaluated concentration range (R^2^ ≥ 0.99). The maximum uncertainty observed for the calibration curves was 1.6 mV. If the calibration curves are compared with the one obtained with the Nernst equation, we can observe that both sensors showed low discrepancies. Deviations in the slope were only 4.6% or lower for BCZY-Pellet sensors and 6.8 or lower for BCZY-Crucible sensors. According to these results, both sensors showed Nernstian behavior, especially in the slopes. In contrast, deviations in the Y-intercept are slightly higher (17.8 and 20.3% of deviation compared with the theoretical value). This deviation implied a maximum error of 28 mV at 0.18 mbar for both types of sensors. This fact can be attributed to electrode phenomena that need to be further studied in future works. The limit of detection was calculated according to [35]. As can be observed, the calculated LOD was 1.6·10^−3^ mbar for BCZY-Pellet and 1.7 ·10^−3^ mbar for BCZY-Crucible. Both sensors had practically the same limit of detection. 

### 3.3. Response Time, Recovery Time and Accuracy

**Figure 8:**
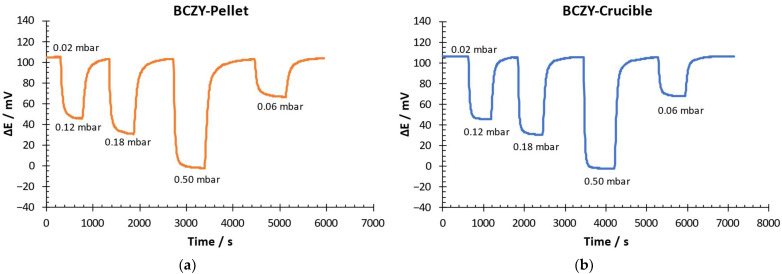
Dynamic response–recovery curve of (**a**) BCZY-Pellet and (**b**) BCZY-Crucible.

Response and recovery times were calculated by measuring a dynamic response and recovery curve. The hydrogen partial pressure at the beginning of the experiment was 0.02 mbar. The potential difference was measured after hydrogen partial pressure changes at 0.12, 0.18, 0.5 and 0.06 mbar. Once the potential difference was stable after each H_2_ partial pressure increment, the initial H_2_ concentration was recovered (0.02 mbar). Response /recovery time was calculated as the time taken for the sensors to achieve 90% of the final potential difference after an increase/decrease of the hydrogen partial pressure, respectively [36,37]. Figure 8 shows the results obtained for both sensors. 

**Figure 9:**
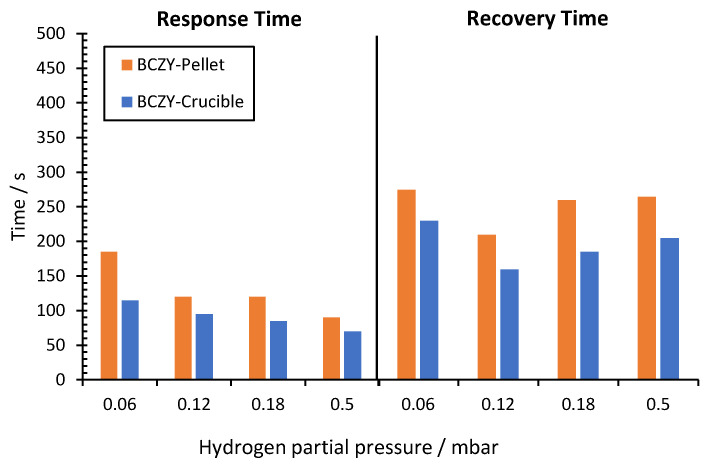
Response and recovery times for different hydrogen partial pressures for BCZY-Pellet and BCZY-Crucible sensors.

As can be seen in Figure 8, both sensors were able to detect each hydrogen concentration change quickly. With these results, the response and recovery times were calculated (see Figure 9).

Response time for BCZY-Pellet sensors when the hydrogen partial pressure increased from 0.02 to 0.06 mbar was 185 s. When the H_2_ partial pressure increment was higher (0.12, 0.18 and 0.5 mbar), these values decreased to around 100 s. Recovery times were between 210 and 275 s. 

For BCZY-Crucible sensors, the response time ranged between 70 and 115 s. It decreased when the hydrogen partial pressure increment was higher. Moreover, recovery times ranged between 160 and 230 s. 

The BCZY-Crucibles sensors showed a 15% lower response and 27% lower recovery times than the BCZY-Pellet sensors, on average, after comparing the two sensors. This fact can be attributed to two different reasons: (i) the area of the platinum electrodes, which can help to achieve the equilibrium between electrodes since the response time quickly, is essentially a property related to the physical characteristics of the electrodes [38]; and (ii) the BCZY-Crucible sensors’ geometry, which can have a better orientation to the gas flow. The effect of the contact angle between the electrode and the gas flow rate is reported in the bibliography [39]. 

**Figure 10:**
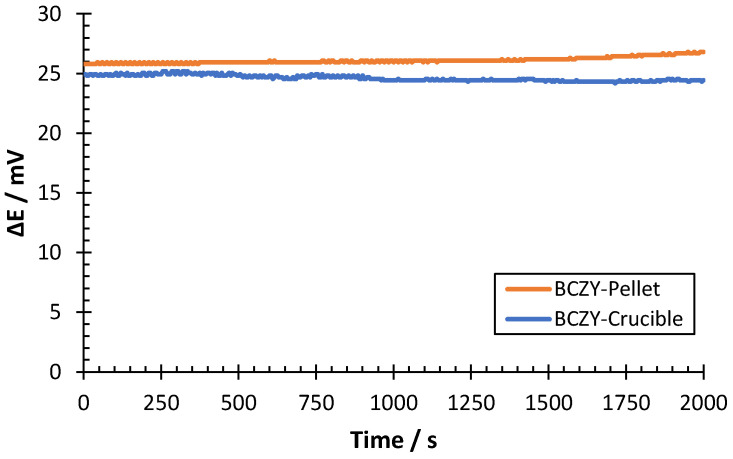
ΔE measurement of a 0.2 mbar H_2_ sample at 500 °C for BCZY-Pellet and BCZY-Crucible sensors.

In another set of experiments, the accuracy of the sensors was determined by measuring a hydrogen sample of 0.2 mbar of H_2_ in Ar. The accuracy was calculated as the discrepancy between the interpolated value in the calibration curve and the nominal partial pressure value. Measurements were performed with both sensors at 500 °C (Figure 10). 

The potential difference shown in Figure 10 remained stable for both sensors during all measurements, with oscillations lower than 1 mV. The average potential difference was interpolated in the calibration curve of each sensor (see Table 2) to determine the discrepancy in the hydrogen partial pressure determination. Results are shown in Table 3.

As can be observed in Table 3, both sensors were able to provide a proper quantification of the hydrogen sample. The uncertainty of the H_2_ measured was 0.003 mbar for both sensors. The maximum discrepancy obtained in the measurements was 4.3%. Thus, both sensors showed discrepancies lower than 5% from the nominal concentration, which indicates good accuracy for the developed sensors. 

The stability of the sensors at high temperatures was studied for 30 days at 500 °C. The variation of the sensors’ responses in this period was lower than 1 mV for the same hydrogen partial pressure. When the solid-state electrolyte was studied after 30 days, no changes were observed in the XRD pattern. In future studies, the long-term stability will be further evaluated.

Obtained results were compared with other sensors obtained in previous works (see Table 4). 

All the sensors developed in previous works [18,19] (see Table 4) were obtained by uniaxial pressure instead of 3D printing. The sensors reported in [18] (both Sr(Ce_0.9_Zr_0.1_)_0.95_Yb_0.05_O_3-α_ and BaCe_0.6_Zr_0.3_Y_0.1_O_3-α_) were evaluated in amperometric configuration. When comparing with the potentiometric sensors constructed in this work, it can be observed that BCZY-Pellet and BCZY-Crucible showed linearity at a wider hydrogen partial pressure range with faster response time. Finally, in reference [19], three different sensors based on BaCe_0.6_Zr_0.3_Y_0.1_O_3-α_ were tested as a function of the sintering temperature and the use of ZnO as a sintering aid. When comparing the potentiometric sensors constructed with 3D printing with those obtained by uniaxial pressure [19], it can be observed that only the sensor with ZnO has a similar behavior. Both sensors showed similar response times, linearity and slopes, obtaining low discrepancies with the Nernst equation. 

Other potentiometric sensors in the bibliography for similar temperatures to those explored in this work are based on yttria-stabilized zirconia (YSZ) with different sensing electrodes, such as CdWO_4_, SnO or MnWO_4_ [40,41,42]. These sensors presented good sensitivities at high hydrogen partial pressure ranges (PH_2_ ≥ 1 mbar), but their response was not Nernstian. In contrast, other authors used similar pellet-shaped perovskite materials (BaCe_0.7_Zr_0.1_Y_0.2_O_3-α_) as electrolytes [43] in Nernstian potentiometric and amperometric sensors. Although both sensors work in a potentiometric mode and present a Nernstian behavior, their linear ranges are complementary (1–100 mbar H_2_ for sensors in [43] and 0.02–0.5 mbar H_2_ in this work). It is worth mentioning that Ba-based ceramics with high cerium and low zirconium contents tend to carbonate at high temperatures [44]. For this reason, the ceramic used in this work, BaCe_0.6_Zr_0.3_Y_0.1_O_3-α_, has the advantage of being more stable against carbon dioxide than the ceramic selected in [43], BaCe_0.7_Zr_0.1_Y_0.2_O_3-α_, since the electrolyte used in this work has lower cerium and higher zirconium content.

## 4. Conclusions

The 3D printed sensors characterized in the present work show a great performance in the evaluated hydrogen concentration range. Both sensors showed a Nernstian behavior in the range from 0.02 to 0.5 mbar. The limit of detection was 1.6·10^−3^ and 1.7·10^−3^ mbar H_2_ in Ar for the BCZY-Pellet and BCZY-Crucible, respectively. Both sensors showed a quick response after a dynamic sequence of increasing and decreasing the H_2_ partial pressure. For BCZY-Pellet sensors, the response time ranged between 90 and 185 s and the recovery time between 210 and 275 s. For the BCZY-Crucible, the response times were 70 to 115 s and the recovery times were 160 to 230 s. The BCZY-Crucible sensors showed 15% lower response and 27% lower recovery times than the BCZY-Pellet sensors, on average. This fact could be attributed to two different reasons: a larger area or a better orientation to the gas flow for BCZY-Crucibles, caused by their geometry. Finally, the accuracy was determined by measuring a sample with a nominal concentration of 0.2 mbar H_2_ for both sensors. The highest discrepancy obtained between the nominal and the measured partial pressure was 4.3%. Therefore, the results obtained with the sensors based on crucible-shaped electrolytes (BCZY-Crucible) are equivalent to those with the pellet-shaped electrolytes (BCZY-Pellet). The change in the geometry (crucible instead of the pellet) is not a parameter that can negatively affect the performance of the sensor, which means that the shape of the electrolyte can be adapted to the particular requirement of each application field. 

In future works, 3D printing will be performed using BCZY ceramic slurry with sintering aids. These additives improve the densification of the electrolyte by using lower temperatures during the sintering process without negatively affecting the performance of the sensors. This way, it is expected to obtain fully dense electrolytes that could enhance the analytical parameters of the sensors.

## Figures and Tables

**Table 1:** Printing parameters used for shaping BCZY-Pellets and BCZY-Crucibles.

	BCZY-Pellet	BCZY-Crucibles
Layer height	0.5 mm	
Print speed	100 mm/s	
Tank pressure	2 bar	
Thickness	1.2 mm	
Printing pattern	Zig-zag	Spiralize contour

**Table 2:** Calibration curves of the sensors, deviation from the Nernst equation and LOD at 500 °C.

Sensor	Calibration Curve	Uncertainty/mV	Correlation Coefficient/R^2^	Slope Deviation/%	Y-Intercept Deviation/%	LOD/mbar
BCZY-Pellet	ΔE (mV) = −34.82·ln(P_H2_ *)−270.84	± 1.6	0.9992	+4.6	+17.8	1.6·10^−3^
BCZY-Crucible	ΔE (mV) = −35.57·ln(P_H2_ *)−276.83	± 1.5	0.9989	+6.8	+20.3	1.7·10^−3^
Nernst **	ΔE (mV) = −33.30·ln(P_H2_ *)−230.06	-	-	-	-	-

* P_H2_ expressed in bar; ** Nernst: theoretical calibration curve obtained using the Nernst equation (see Equation (1)) in the experimental conditions used for the measurements.

**Table 3:** Accuracy of the sensors was evaluated with 0.2 mbar hydrogen sample at 500 °C.

T/°C	Sensor	Average ΔE/mV	H_2_ Measured/mbar	Discrepancy/%
500	BCZY-Pellet	26.093 ± 0.502	0.198 ± 0.003	−1.0
	BCZY-Crucible	24.617 ± 0.500	0.209 ± 0.003	+4.3

**Table 4:** Comparison of the analytical parameters of hydrogen sensors obtained in previous works with the sensors proposed in this work.

Material	Geometry/Shaping Technology	Type of Sensor	Sensing Range (mbar)	Response Time (s)	Ref.
Sr(Ce_0.9_Zr_0.1_)_0.95_Yb_0.05_O_3- α_	Pellet/Uniaxial pressure	Amperometric	0.2–0.8	~480	[18]
BaCe_0.6_Zr_0.3_Y_0.1_O_3-α_	Pellet/Uniaxial pressure	Amperometric	0.06–0.2	~240–360	[18]
BaCe_0.6_Zr_0.3_Y_0.1_O_3-α_(Sintered at 1400 °C)	Pellet/Uniaxial pressure	Potentiometric	0.02–0.5	~100	[19]
BaCe_0.6_Zr_0.3_Y_0.1_O_3-α_(Sintered at 1650 °C)	Pellet/Uniaxial pressure	Potentiometric	0.02–0.5	~100	[19]
BaCe_0.6_Zr_0.3_Y_0.1_O_3-α_-ZnO	Pellet/Uniaxial pressure	Potentiometric (Nernstian)	0.02–0.5	~100	[19]
BaCe_0.6_Zr_0.3_Y_0.1_O_3-α_(BCZY-Pellet)	Pellet/3D Printing	Potentiometric (Nernstian)	0.02–0.5	~100–185	This work
BaCe_0.6_Zr_0.3_Y_0.1_O_3-α_(BCZY-Crucible)	Crucible/3D Printing	Potentiometric (Nernstian)	0.02–0.5	~70–115	This work

## Data Availability

The data is contained within the article.

## Supplementary Materials

The following supporting information can be downloaded at: https://www.mdpi.com/article/10.3390/s22249707/s1, Figure S1: Image of the 3D printed samples.
