# Supplementary figures and images for: Potentiometric Hydrogen Sensor with 3D-Printed BaCe0.6Zr0.3Y0.1O3-α Electrolyte for High-Temperature Applications
Source: Sensors (Basel). 2022 Dec 11;22(24):9707. doi: 10.3390/s22249707 (PMC9785787; doi:10.3390/s22249707)

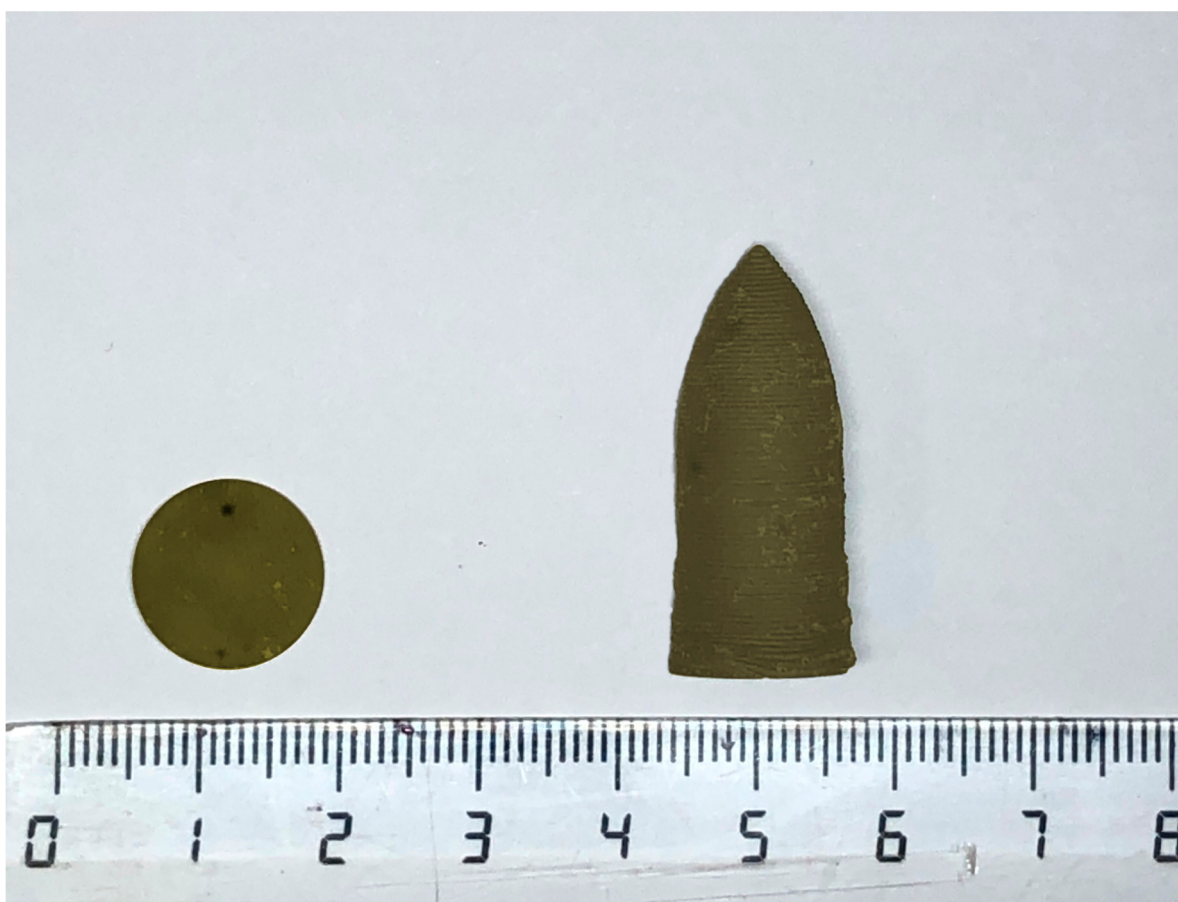

**Figure S1.** Image of the 3D printed samples.

Supplement: Supplementary file 1 [file sensors-22-09707-s001.zip › sensors-2066580-supplementary.pdf]
